# Three-Dimensional Intraoral Scanners in Oral Soft Tissue Diagnosis: A Narrative Review and Conceptual Outlook

**DOI:** 10.7759/cureus.100113

**Published:** 2025-12-26

**Authors:** Maria Myrto Solomou, Yiannis Pistolas, Alexandros Theodosiou, Andreas S Panayides, Smaragda Diamanti

**Affiliations:** 1 Oral Medicine, European University Cyprus, Nicosia, CYP; 2 Dentistry, European University Cyprus, Nicosia, CYP; 3 Computer Science, University of Cyprus, Nicosia, CYP; 4 Videomics, CYENS Centre of Excellence, Nicosia, CYP

**Keywords:** artificial intelligence, digital diagnostics, intraoral scanner, oral soft tissue lesions, teledentistry, three-dimensional imaging

## Abstract

Oral soft tissue lesions encompass a wide spectrum of conditions, from benign alterations to potentially malignant disorders and malignancies. Accurate diagnosis and continuous monitoring are vital for timely intervention and improved prognosis. Current diagnostic techniques, visual and tactile examination, two-dimensional (2D) clinical photography, and histopathological analysis when required, remain indispensable but have notable limitations. Visual inspection is subjective, 2D photography cannot reliably capture volumetric or surface-texture changes, and biopsies are invasive and impractical for routine follow-up. This narrative review synthesizes existing evidence on intraoral scanning (IOS) technologies in dentistry and explores their potential application in the documentation and monitoring of oral soft tissue lesions. A narrative literature review was conducted across PubMed, Scopus, IEEE Xplore, and Embase databases up to September 20, 2025. Search terms included “intraoral scanner”, “oral soft tissue”, “oral lesions”, “3D imaging”, and “teledentistry”. Studies describing IOS applications in general dentistry, specialty practice, or soft-tissue imaging were included. Relevant findings were thematically analyzed to assess the feasibility of IOS in oral medicine. IOS offers objective, reproducible, and non-invasive three-dimensional (3D) data acquisition, enabling volumetric lesion monitoring, digital record-keeping, telemedicine integration, and potential artificial intelligence (AI)-based analysis. However, technical limitations persist, including motion artifacts, saliva interference, restricted depth penetration, and difficulty capturing highly mobile mucosa. Importantly, no studies to date have directly evaluated IOS for diagnosing oral soft tissue lesions. Integrating high-resolution 3D IOS into oral medicine could enable accurate, repeatable, and non-invasive documentation of soft tissue lesions, supporting longitudinal assessment, diagnosis, patient education, and teleconsultation. Nonetheless, further research addressing training requirements, cost-effectiveness, soft-tissue imaging optimization, and scanner resolution is essential before clinical adoption.

## Introduction and background

The use of digital technologies in dentistry has revolutionized patient care by substituting more precise, efficient, and patient-centered procedures for antiquated analog techniques [1]. In this changing digital world, oral soft tissue lesions continue to pose a serious diagnostic challenge. From benign reactive lesions to potentially harmful disorders and cancers, these conditions cover a wide spectrum. For selected oral lesions that require follow-up, such as oral potentially malignant disorders (OPMDs), precise diagnosis, documentation, and longitudinal monitoring are crucial for effective intervention and better patient outcomes [2,3]. This process is complicated, though, by the variety of clinical manifestations of these lesions, as well as minute morphological changes in size, shape, or surface texture that are frequently challenging to detect precisely. These difficulties are made worse by examiner subjectivity and variations in lighting during evaluation [3]. Visual and tactile examination, clinical photography, and, if required, biopsy for histopathological confirmation are standard components of modern diagnostic procedures [3]. These techniques are still essential, but they have drawbacks: clinical examination is subjective and prone to inter-examiner variability, traditional two-dimensional (2D) photography cannot reliably capture volumetric or surface-texture changes over time, and biopsies are invasive and inappropriate for routine follow-up [4].

**Figure 1:**
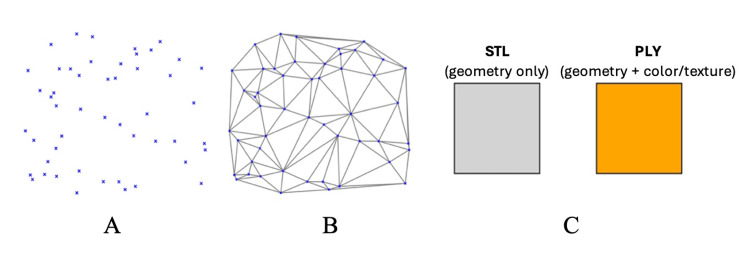
From point clouds to digital file formats in intraoral scanning. (A) Point cloud (discrete 3D coordinates captured by the scanner). (B) Polygon mesh created by triangulating the point cloud into a continuous surface. (C) Exported file formats: STL (geometry only) and PLY (geometry + color/texture) [3-8].

Three-dimensional (3D) intraoral scanning (IOS) technology presents a viable digital substitute in this regard. In order to fully digitize oral anatomy, intraoral scanners are medical devices that record the 3D geometry of teeth and the soft tissues around them. The scanner takes several pictures of the dento-gingival tissues using structured light or laser projection. These pictures are then converted into point clouds, which are sets of distinct spatial data points (x, y, z) that show the exact location of the scanned surface. The accompanying software then triangulates these point clouds into continuous polygonal meshes, creating high-fidelity virtual representations of the oral environment. The resulting datasets are saved in standardized file formats such as PLY (polygon file format), which can also preserve surface color and texture, or STL (stereolithography), which only records geometric information (Figure 1). These features have already made 3D intraoral scanners indispensable for implant planning, orthodontics, and restorative dentistry [5-8].

In implantology, orthodontics, and restorative dentistry, intraoral scanners have received extensive validation. They enable computer-aided design (CAD)/computer-aided manufacturing (CAM) prosthetics, aligner manufacture, and implant surgical guides by providing digital impressions with a precision of <20 µm [8,9]. These applications confirm that IOS can reliably replace conventional impressions in hard tissue contexts. Modern devices integrate three core components: a handheld wand containing optical sensors (laser or structured light sources) with motion-tracking accelerometers, a processing unit that reconstructs 3D meshes from 2D images using simultaneous localization and mapping (SLAM) algorithms, and software interfaces for visualization, measurement (±5-20 µm accuracy), and export into standardized file formats, such as STL and PLY [1,10].

However, as far as we are aware, no research has explicitly looked into the use of 3D intraoral scanners for the diagnosis and recording of soft tissue lesions in the mouth. In terms of quantitative, repeatable, and non-invasive lesion monitoring in particular, this constitutes a substantial gap in the current diagnostic toolkit for oral medicine. In order to encourage further research and integration into oral medicine workflows, this narrative review presents for the first time the idea of using 3D intraoral scanners as a diagnostic and monitoring tool for oral soft tissue lesions. It does this by describing the clinical justification, expected advantages, and expected drawbacks.

## Review

Materials and methods

Search Strategy

A comprehensive literature search was performed in PubMed, Scopus, Institute of Electrical and Electronics Engineers (IEEE) Xplore, and Embase library up to the 20th of September 2025. IOS technology, clinical oral soft tissue context, and diagnostic and workflow applications were the three domains that were integrated into the Boolean strategy. To find additional appropriate research, the reference lists of all included papers and relevant reviews were also manually examined. The full search string was as follows: (“intraoral scanner” OR “intraoral scanning” OR “digital impression” OR “3D optical scanning” OR “structured light scanning” OR “triangulation laser” OR “confocal imaging” OR “chromatic confocal” OR “optical coherence tomography” OR “hyperspectral imaging”) AND (“oral mucosa” OR “oral soft tissue” OR “oral lesions” OR “oral pathology” OR “oral potentially malignant disorders” OR “OPMD” OR “oral cancer”) AND (“diagnostic accuracy” OR “precision” OR “trueness” OR “reproducibility” OR “surface texture capture” OR “lesion monitoring” OR “digital workflow” OR “teledentistry” OR “telemedicine” OR “remote consultation” OR “artificial intelligence” OR “AI”).

Eligibility Criteria

Peer-reviewed English-language studies that examined IOS technologies in relation to diagnostic precision, accuracy, repeatability, or soft tissue applications were eligible for inclusion. Studies addressing telemedicine integration, digital workflows, artificial intelligence (AI), or 3D scanning technologies in IOS were considered, including clinical, engineering, review, and experimental reports relevant to diagnostic applications. Studies unrelated to imaging or diagnostic applications, as well as non-scientific materials (e.g., conference abstracts, newsletters, opinion pieces), were excluded.

Study Selection and Synthesis

Article selection followed the predefined scope of the review. Two authors independently screened the titles and abstracts of retrieved records for relevance. Full-text assessment was subsequently performed by the same reviewers. In cases of disagreement regarding inclusion, consensus was reached through discussion, and when necessary, a third author acted as an arbiter.

We recorded database yields and provided a simple flow summary: (n) records identified=135 (PubMed=33, Embase=27, Scopus=38, IEEE Xplore=21, reference list screening=16), duplicates removed=32, titles/abstracts screened=103, full texts assessed=83, and a total of n=50 articles were included in the final synthesis.

Data extraction was performed qualitatively, without the use of standardized extraction forms, focusing on reported technical characteristics, accuracy metrics, clinical applicability, and limitations of IOS technologies. Themes were derived iteratively through narrative synthesis, grouping studies according to recurring clinical and technological concepts rather than through a formal coding framework.

Data were narratively extracted and thematically synthesized into four domains: (1) existing IOS dental applications, (2) soft tissue capture evidence, (3) telemedicine and lesion monitoring applications, and (4) limits and future directions.

No statistical pooling, meta-analysis, or quantitative synthesis was attempted, as the included studies were highly heterogeneous in design, outcomes, and measurement methodologies, making quantitative aggregation inappropriate for the objectives of this narrative review.

Given the narrative and exploratory nature of this review and the heterogeneity of included study designs (engineering studies, clinical validation studies, observational reports, and reviews), a formal risk-of-bias assessment using standardized tools was not performed. Instead, methodological limitations were reported, and study design constraints were considered qualitatively during interpretation and are discussed where relevant in the Results and Discussion sections.

Results

This narrative review identified a diverse but fragmented body of literature on IOS technologies relevant to diagnostic applications in oral medicine. With little investigation of mucosal or lesion-specific imaging, the majority of published work has been on restorative and orthodontic purposes. The results are categorized thematically into four domains for ease of understanding: (1) soft tissue capture evidence, (2) lesion monitoring and telemedicine applications, (3) technical barriers mentioned, and (4) research gaps.

Evidence for Soft Tissue Capture

Although IOS technologies were primarily developed for hard tissue imaging, several optical principles have been applied to extend their use to mucosal surfaces.

Triangulation laser scanning, employed in devices such as 3Shape TRIOS and iTero Element, projects laser stripes that are interpreted by dual cameras via trigonometric parallax, achieving precision in the range of 7-15 µm for enamel margins and fissures [9]. However, this method shows reduced reliability in dark composites, reflective surfaces, and saliva-rich regions [11,12].

Confocal imaging systems, such as Medit i700, apply chromatic depth discrimination and can capture undercuts without the need for reflective powder coatings that were required in earlier devices. While this simplifies acquisition and preserves natural tissue color, depth penetration remains restricted to 4-6 mm, which limits performance in saliva-rich or mobile mucosal environments [13].

Active wavefront sampling (AWS), used in the 3M Lava C.O.S./True Definition scanners and exemplified by the Planmeca Emerald S, reconstructs 3D data via an off-axis rotating aperture and an oscillating lens system that captures multiple focal planes, enabling rapid full-arch acquisition in under a minute. Like other optical scanners, it can be affected by reflection artifacts from metallic restorations [14].

Finally, optical coherence tomography (OCT) systems, which employ 1,300 nm near-infrared (NIR) light to generate subsurface tissue images, show promise for early lesion characterization and enamel porosity mapping [15,16]. However, due to their small field of view and lengthy multi-volume acquisitions, intraoral OCT often requires tens of minutes for comprehensive coverage, limiting routine chairside use [17]. Collectively, these technologies demonstrate that IOS can visualize gingival margins and selected mucosal contours, particularly in relatively immobile and keratinized areas such as the attached gingiva and hard palate, with acceptable reproducibility, but systematic validation for diagnostic lesion imaging is still lacking. To better illustrate these differences, the main optical technologies are compared side by side, highlighting their performance characteristics, advantages, and limitations in relation to soft tissue applications (Table 1).

**Table 1:** Performance characteristics of intraoral scanner technologies in soft tissue imaging. Summary of published evidence on the optical principles and diagnostic performance of intraoral scanners used for soft-tissue capture, including confocal imaging, triangulation laser/structured light, active wavefront Sampling (AWS), and optical coherence tomography (OCT) [9,11-17]. N/A = Not applicable; AWS = Active wavefront sampling; OCT = Optical coherence tomography Soft tissue suitability score (1-5): a qualitative, expert-informed score reflecting the relative suitability of each IOS technology for oral soft tissue imaging, based on narrative synthesis of published evidence regarding surface accuracy, stability in moist environments, motion sensitivity, depth penetration, and color/texture capture. This score is not derived from a formal quantitative calculation but is intended to provide a comparative overview for conceptual purposes.

IOS Technology	Example Devices	Resolution Accuracy	Color/Texture Capture	Depth Penetration	Speed	Soft Tissue Suitability Score (1–5)	Key Advantages	Key Limitations
Triangulation Laser/Structured Light	iTero Element, Dental Wings	±7–15 µm	Good–Excellent (depends on reflectivity)	2-4 mm	Fast (arch in ~1 min)	4/5	Very high surface accuracy, good color on keratinized tissue	Struggles with moist/dark surfaces (low reflectivity), glare sensitivity
Confocal Imaging	Medit i700, 3Shape TRIOS 4, Carestream CS 3700	±10–20 µm	Excellent, true color	4-6 mm	Moderate (full arch 1-2 min)	4/5	Good for undercuts, moist surfaces, powder-free, stable in variable light	Limited penetration for deep lesions, moderate learning curve
AWS	Planmeca Emerald S	±15–25 µm	Moderate	3-5 mm	Very fast (0.5-2 sec per arch)	3/5	Extremely quick scanning, simple workflow	Prone to metallic/moisture artifacts, moderate color fidelity
OCT	Research prototypes, NIR-OCT systems	±5–10 µm	N/A (grayscale subsurface imaging)	1-2 mm subsurface	Very slow (8-12 min per arch)	2/5	Subsurface detail, early lesion detection potential	Impractical scan times, small field of view, research-only

Applications in Lesion Monitoring and Telemedicine

Once feasibility and accuracy considerations are addressed, the potential clinical value of IOS extends beyond chairside documentation to longitudinal monitoring and remote consultation.

Emerging evidence supports the use of IOS for reproducible documentation and longitudinal monitoring of oral soft tissues and dental structures. High-resolution scans have been shown to capture soft tissue morphology in anatomically stable regions and to enable reproducible follow-up through superimposition of sequential datasets, as demonstrated in studies on palatal soft tissue anatomy and dental wear [4,18,19]. However, direct evidence supporting the use of IOS for monitoring pathological oral soft tissue lesions is currently lacking. IOS provides objective, quantitative, and reproducible 3D models that measure surface area, volume, and texture, allowing clinicians to assess morphological changes over time using measurable parameters [4,19]. Superimposed scans can reveal early alterations, such as volumetric increase, contour irregularities, or border changes, that may precede visible progression, a critical advantage for potentially malignant disorders where early intervention improves prognosis [18,19]. Being contactless and non-invasive, IOS may support closer follow-up between clinically indicated biopsies by enabling comfortable, low-risk monitoring, potentially improving patient compliance; however, biopsy remains essential for definitive diagnosis [20]. The resulting 3D datasets enhance patient-clinician communication and interdisciplinary collaboration by providing intuitive visualizations of lesion morphology and progression, supporting patient understanding and adherence [21]. Moreover, these digital records can be securely stored, archived, and transmitted for remote consultation. While traditional teledentistry has relied on 2D images or video, often limited by lighting and lack of volumetric data [22], IOS offers objective, high-resolution 3D information. Studies have demonstrated strong agreement between IOS-based teledental assessments and conventional clinical examinations in pediatric populations [23] and validated its use for epidemiological caries diagnosis [24]. Additional evidence confirms that teledentistry platforms can achieve diagnostic outcomes comparable to in-person assessments [25,26]. Although none have yet addressed mucosal lesions, these findings establish the feasibility of extending IOS to remote oral medicine. This approach could be transformative for patients in remote or resource-limited regions, where delayed access to oral medicine specialists often leads to poorer outcomes. IOS-based teleconsultations would allow community dentists to capture standardized 3D models and transmit them to experts for rapid triage, reducing referral delays, unnecessary travel, and improving early detection. De-identified scans could also enrich tele-education platforms, supporting standardized training in digital diagnostics and oral medicine. Despite its potential, several challenges must be addressed before widespread implementation. The management of large 3D datasets requires secure, encrypted transfer and long-term storage compliant with data protection regulations such as GDPR and HIPAA. Bandwidth limitations in areas with poor internet connectivity may also hinder real-time teleconsultations, while the cost of equipment and the need for user training could restrict adoption in resource-limited settings [20,21]. However, the primary barrier remains the lack of clinical validation of intraoral scanning for oral soft tissue lesions. Only after feasibility, accuracy, and reproducibility are demonstrated in oral medicine could overcoming technical and logistical barriers enable IOS to become an integral component of telemedicine, supporting remote consultation, longitudinal monitoring, and the future development of AI-driven tools for detecting early morphological indicators of malignancy [7,20].

Reported Limitations and Technical Barriers

Despite these advantages, IOS faces persistent technical challenges. Motion artifacts exceeding 200 µm can disrupt alignment algorithms, particularly in mobile tissues such as the tongue and buccal mucosa [27]. Saliva and blood cause specular reflections and stitching errors, while homogeneous mucosa lacks sufficient landmarks for stable tracking [9,28]. AWS devices remain highly sensitive to reflective interference, and OCT is still impractical for routine chairside use [14,17]. Capturing fine surface texture and subtle color variations in soft tissues also remains difficult, with differences in calibration and intraoral lighting reducing reproducibility across devices [4,9].

Current IOS protocols are optimized for dentition and prosthodontics, and no standardized guidelines exist for soft tissue imaging regarding angulation, distance, lighting, or data processing, leaving results vulnerable to inter-operator variability [1,20]. Mitigation strategies include video-rate scanning (≥60 fps) to minimize motion artifacts [10], titanium dioxide (TiO₂) powder sprays to enhance surface contrast [9], and anti-fog tips with absorbent pads to maintain optical clarity [8]. However, no commercial system reliably scans the buccal mucosa, floor of the mouth, or tongue, where dynamic deformation, saliva pooling, and absence of fixed reference points cause stitching errors beyond algorithmic tolerance [12,27]. Additional factors, such as low surface contrast [9], patient movement [29], and lesion bleeding [30], further compromise stability and accuracy. Optimal results often require operator experience, as inexperienced users may produce incomplete or distorted scans [8]. Reliable imaging currently appears limited to lesions on non-movable, keratinized mucosa, such as attached gingiva or hard palate, where proximity to stable landmarks supports accurate registration [4]. Finally, inconsistencies in the methods used to assess scanner accuracy complicate inter-study and inter-device comparisons.

Accuracy metrics and influencing factors: Beyond acquisition feasibility, the clinical reliability of intraoral scanners depends on how accurately and reproducibly surface data can be captured and compared over time.

With the advent of newer generations of IOS, variations in accuracy between devices have been observed [31]. Because STL files are constructed from virtual 3D coordinates, accuracy is assessed using different approaches. According to the International Organization for Standardization (ISO 5725-1), accuracy comprises two elements: trueness, the closeness of agreement between the mean value of repeated measurements and a reference standard, and precision, the degree of agreement among repeated measurements under defined conditions [32]. In dental research, the mean absolute deviation (MAD) has traditionally been used, while the root mean square (RMS) method has more recently been introduced to calculate absolute point-to-point deviations across the scanned region [33]. Multiple variables influence IOS accuracy. These include hardware-related factors such as differences in data acquisition technologies [34], software processing algorithms [35], and object characteristics such as model material [36], restorative materials [37], span length [38], and additional surface treatments [39]. Clinical conditions, including moisture, bleeding, and restricted access, further affect reproducibility and trueness [40]. Additional telemedicine-related challenges, including secure transfer and long-term storage of large 3D datasets, bandwidth limitations, cost, and training requirements, have been discussed above. These factors remain important considerations for the broader implementation of IOS-based telemedicine in oral medicine [1,20,21].

Research Gaps

This review identified significant gaps in the literature. Despite extensive validation of IOS in hard tissue applications, no peer-reviewed studies directly assess its use in oral soft tissue lesion documentation, diagnosis, or monitoring. There are no standardized protocols for lesion scanning, volume quantification, or longitudinal follow-up. Accuracy reporting remains inconsistent, with some studies using MAD and others RMS without clarity on their clinical implications [32,33]. The absence of lesion-specific datasets further hampers the development of AI models capable of detecting morphological predictors of malignant transformation [7]. The creation of sizable, standardized datasets of 3D lesion scans may facilitate studies on risk assessment, progression trends, and automated diagnostic instruments.

Table 2 offers a comparative summary of the expected advantages of intraoral scanning for oral soft tissue lesions, along with existing restrictions and adoption hurdles, in order to compile the possible clinical consequences of this procedure.

**Table 2:** Potential benefits and challenges of using intraoral scanners (IOS) for oral soft tissue lesion documentation and monitoring. Summary of key domains where IOS technology may influence oral soft tissue diagnostics, clinical workflows, and research. While 3D intraoral scanning enables non-invasive, reproducible lesion visualization and supports telemedicine and AI-driven innovation, current limitations include motion artifacts, lack of standardized soft tissue scanning protocols, and technical or interoperability constraints [4,7-10,12,14,17,20-24,27,28-30].

Domain	Potential Benefits	Challenges/Limitations
Diagnostic Accuracy & Monitoring	• High-resolution, reproducible 3D models of lesion morphology (surface area, volume, texture). • Enables superimposition of sequential scans to detect subtle morphological changes earlier than clinical exam or 2D photography. • Non-invasive alternative to repeated biopsies, supporting closer follow-up.	• Motion artifacts (>200 µm) in mobile tissues (tongue, buccal mucosa). • Saliva, bleeding, and specular reflections distort accuracy. • Low surface contrast in homogeneous mucosa provides poor tracking features. • Currently limited to lesions on non-movable, keratinized mucosa (palate, attached gingiva); not reliable for tongue, floor of mouth, or buccal mucosa.
Clinical Workflow & Patient Care	• More comfortable for patients compared to invasive monitoring. • Enhances patient engagement through visual 3D models. • Supports evidence-based decisions (e.g., biopsy timing).	• Lack of standardized protocols for lesion scanning (angulation, distance, lighting). • Operator learning curve: inexperienced users may produce incomplete or distorted scans. • Powdering required in earlier systems distorted color and added complexity. • Procedural instability in saliva-rich areas without mitigation (e.g., anti-fog tips, suction, powders).
Telemedicine & Collaboration	• Secure digital records can be stored, archived, and shared for remote consultation. • Feasible integration into teledentistry platforms. • Potential to reduce referral delays and support triage in underserved areas. • Supports interdisciplinary collaboration and education (tele-education).	• Large file sizes require encrypted transfer and compliant long-term storage (GDPR, HIPAA). • Bandwidth constraints in low-connectivity regions may limit synchronous use. • Interoperability challenges across platforms.
Research & Innovation	• Foundation for lesion-specific 3D databases. • Potential for AI-driven diagnostic algorithms (early malignancy detection). • Can support global oral cancer prevention strategies through standardized monitoring.	• Absence of clinical trials directly validating IOS for soft tissue lesion diagnosis. • No standardized methods for depth or volume quantification. • OCT impractically slow (8-12 min/arch). • High device cost and limited availability in resource-poor settings. • Heavy data management requirements (storage, IT infrastructure).

Discussion

The findings of this narrative review, summarized in Tables 1-2, highlight both the opportunities and limitations of current IOS technologies for documenting oral soft tissue lesions. Although triangulation, confocal, AWS, and OCT represent distinct optical principles, evidence consistently indicates that confocal systems are most promising for mucosal surfaces. Their ability to capture undercuts without powdering, preserve tissue color, and maintain stability under variable lighting makes them particularly suited to oral lesion imaging [13]. In contrast, triangulation and structured-light scanners achieve high accuracy on reflective hard tissues but perform poorly on moist or low-contrast mucosa [11,12], while AWS prioritizes acquisition speed over diagnostic reliability [14]. OCT provides unique subsurface information and potential for early lesion characterization [15,16], yet long scan times and a limited field of view constrain clinical use [17].

Clinically, IOS may serve as a non-invasive adjunct for lesion monitoring, especially in areas with stable mucosa such as the palate or attached gingiva. More mobile or moisture-prone regions, including the lateral tongue and floor of the mouth, remain challenging and require refinement of acquisition protocols and alignment algorithms. Volumetric monitoring may represent a future adjunct for surveillance in high-risk patients with OPMDs; however, no studies to date have demonstrated that IOS-detected volumetric or surface changes predict malignant transformation, and this hypothesis requires prospective validation [4,18].

Accuracy assessment also warrants standardization, as MAD and RMS are often used interchangeably despite differing implications. RMS emphasizes larger errors and may be more relevant for subtle mucosal changes; future research should establish clinically meaningful thresholds for soft-tissue applications [40].

Equally critical is demonstrating integration into real-world care pathways. Telemedicine offers a particularly impactful application in underserved regions, enabling triage and earlier access to specialist expertise [23,24]. Coupled with AI, IOS could support semi-automated detection of lesion progression and morphological predictors of malignant transformation [7,20].

Clinical Implications

In daily practice, the integration of intraoral scanners into the documentation of soft-tissue lesions can provide several immediate benefits. Beyond the research and technical aspects, clinicians may gain more accurate and reproducible monitoring of lesion changes over time, with evidence showing that intraoral scans can reliably detect morphological changes and volumetric alterations that may be missed by 2D methods [18,19]. Digital 3D records also support enhanced patient communication, as high-resolution visual models improve patient understanding and engagement [21]. Moreover, scanners open the door to teledentistry, where 3D datasets can be securely shared for remote consultations, with studies confirming the feasibility of iOS-based tele-assessments and lesion triage [22]. Finally, these datasets are AI-ready, enabling future automated lesion detection as larger repositories emerge [1,20].

Study Limitations

As a narrative review, this study has inherent limitations and does not claim to exhaust all available evidence. Nevertheless, by incorporating literature from telemedicine, biomedical optics, and dentistry, it captures the most relevant clinical and technological perspectives. Although IOS has not yet been tested in clinical trials for oral lesion imaging, this gap underscores the novelty of the concept and the importance of early conceptualization in guiding future research. The absence of standardized scanning protocols and debate regarding optimal accuracy metrics reflects not methodological flaws but the developmental stage of this emerging field. Such variability highlights opportunities for targeted methodological refinement. Additionally, many existing validation studies are industry-funded, introducing potential publication bias, as positive outcomes are more likely to be reported. Independent, peer-reviewed investigations are therefore essential to establish the clinical reliability and generalizability of IOS applications in soft-tissue lesion documentation.

Future Directions

Based on this review, the integration of IOS into oral medicine should progress through a staged pathway from feasibility to clinical adoption. Initial pilot studies are needed to establish optimal scanning protocols for various lesion types and anatomical sites, assessing IOS accuracy in capturing morphology, surface texture, and color relative to calibrated photography or physical measurements [4,18]. These studies should also address real-world factors, motion artifacts, saliva pooling, and bleeding that affect reproducibility [27]. Subsequent validation trials must determine diagnostic accuracy, sensitivity, and specificity for benign and potentially malignant conditions [19], while longitudinal monitoring could reveal whether volumetric or shape-based changes predict malignant transformation [18]. Standardized acquisition protocols and lesion-scanning modes, developed collaboratively across specialties, will ensure comparability and reliability [20]. Advances in artificial intelligence may enable automated lesion recognition and triage from large 3D datasets [7]. Combining IOS with multispectral imaging, optical coherence tomography, or radiomics could yield multimodal diagnostic frameworks that capture both structural and biochemical features [15]. Finally, standardized metrics for lesion depth and volume, alongside implementation studies on cost-effectiveness, patient acceptance, and training, will be essential for successful clinical integration [8,10,20].

## Conclusions

For the first time, the idea of documenting, diagnosing, and tracking oral lesions utilizing 3D intraoral scanning is presented in this study. By enabling high-resolution, consistent, and non-invasive capture of lesion morphology, 3D scanning provides the potential to solve major limitations of current approaches, particularly in longitudinal follow-up and early change detection. Even though there are still procedural, logistical, and technical obstacles to overcome, the quick development of iOS technology and the increased focus on digital dentistry foster an atmosphere that promotes innovation in oral medicine. Utilizing this technology could improve interdisciplinary collaboration and patient communication, aid in the development of AI diagnostic tools, and increase diagnosis accuracy. Standardized scanning methods, integration with current diagnostic systems, and clinical validation trials are needed to achieve this. In low-resource environments, cost and access continue to be major obstacles, and resolving them is crucial to ensuring fair implementation.
